# A Network of Anomalies Prompting VACTERL Workup in a Trisomy 21 Newborn

**DOI:** 10.7759/cureus.21290

**Published:** 2022-01-16

**Authors:** Trenton Reinicke, Christina L Costantino, Danyon J Anderson, Jacqueline Tran, Cornelia Griggs

**Affiliations:** 1 Department of Clinical and Translational Epidemiology, Massachusetts General Hospital, Boston, USA; 2 Department of Surgery, Massachusetts General Hospital, Boston, USA; 3 School of Medicine, Medical College of Wisconsin, Wauwatosa, USA; 4 Department of Surgery, Massachusetts General Hospital, Harvard Medical School, Boston, USA

**Keywords:** ventricular septal defect, atrioventricular canal defect, hirschsprung’s disease, vascular ring, laryngeal cleft, tracheoesophageal fistula, esophageal atresia, trisomy 21

## Abstract

VACTERL (vertebral defects, anal atresia, cardiac defects, tracheoesophageal fistula, renal anomalies, and limb abnormalities) association is a condition defined by having at least three of the following congenital malformations: vertebral defects, anal atresia, cardiac defects, tracheoesophageal fistula, renal anomalies, and limb abnormalities. While diagnosing the VACTERL association is rare, the conditions that make up the VACTERL core-component features among other congenital abnormalities are even more unique. We present a case of a 34-week-old premature infant with trisomy 21 in addition to esophageal atresia, tracheoesophageal fistula, laryngeal cleft, vascular ring, Hirschsprung’s disease, atrioventricular canal defect, ventricular septal defect, and other related conditions diagnosed at birth. To our knowledge, this case represents the first of its kind in relation to the constellation of anomalies diagnosed in one individual at birth of which may or may not be related to Down syndrome, and the associated interventions necessary to continue postnatal living.

## Introduction

Down syndrome (DS), or trisomy 21, is a genetic disorder caused by the presence of a third chromosome 21. Patients diagnosed with DS have a higher frequency of congenital malformations compared to the general population, in which nearly 40% of patients with DS harbor at least one congenital malformation while having multiple aberrations is much less common [1]. DS typically presents with both constant and diversified clinical features that can include increased risk for congenital heart defects, gastrointestinal abnormalities, and hematologic abnormalities [2]. Some of the DS-associated anomalies that are relevant to this case are further discussed below.

Congenital heart disease (CHD) is a complication that is found in up to 50% of newborns with DS [3], while only impacting 0.3% of newborns without DS [4]. Atrioventricular septal defect (AVSD) is the most common type of CHD among those with DS, followed by a ventricular septal defect (VSD) [3]. Atrioventricular (AV) canal and VSD occur when oxygen-rich blood from the left atrium or ventricle, respectively, mixes with oxygen-deficient blood in the right atrium or ventricle due to communication(s) in the septum of the heart. Complications of AVSD include increased pressure in the right ventricle causing volume overload, pulmonary hypertension, and heart failure [5]. Thus, prompt diagnosis and surgical treatment are essential to maintaining the neonatal quality of life.

Hirschsprung’s disease (HD), also called congenital aganglionic megacolon, is an associated gastrointestinal complication found in 1% to 3% of infants with DS [6]. HD occurs when the enteric nervous system is missing from the end of the bowel, creating aganglionic intestinal segments preventing normal peristaltic and defecation reflexes resulting in a functional occlusion [3]. A pull-through or colostomy is critical following diagnosis to prevent irreversible complications and death that may result.

Congenital esophageal atresia (EA) and tracheoesophageal fistula (TEF) are rare conditions that often occur synchronously and lead to chronic respiratory and digestive issues due to aberrant development and connections of the esophagus and trachea during intrauterine life [7]. EA with or without TEF has been associated with DS but is shown to occur in less than 1% of infants with the condition [6]. More than 50% of EA/TEF patients have other associated cardiac, renal, and/or musculoskeletal anomalies, in which EA/TEF often occurs as part of several other syndromes and associations [8]. EA/TEF represents one of the core components of the VACTERL (vertebral defects, anal atresia, cardiac defects, tracheoesophageal fistula, renal anomalies, and limb abnormalities) association and is seen in 10% to 30% of cases [8]. For this reason, patients with EA/TEF are often assessed for VACTERL association. However, the etiology of the VACTERL association is still unclear as the combination of EA/TEF and other associated anomalies phenotypically resemble that of other known syndromes such as CHARGE (coloboma, heart defect, atresia choanae, retarded growth and development, genital hypoplasia, ear anomalies/deafness), Feingold, and 22q11 deletion syndromes [8]. Thus, it is often hard to discern between what is independently VACTERL in nature or another syndrome. Given that only 0.20% of patients with DS have major anomalies affecting three organs [2], the VACTERL association should be of concern for patients with DS.

Here, we present a rare case of a 34-week-old premature male newborn with DS and a combination of rare congenital abnormalities affecting multiple organ systems prompting VACTERL workup.

This case was presented at the Boston Surgical Society Case of the Year Competition 2021 on March 2, 2021, by Christina L. Costantino, MD.

## Case presentation

A 34-week-old premature male infant was born by cesarean section (C-section) with low birth weight (2.15 kg) to a gravida 2, para 2 (G2P2) 41-year-old mother. The mother’s medical history includes known advanced maternal age (AMA), hypothyroidism, and negative prenatal screens at hospital one (H one). The patient was diagnosed with trisomy 21 by amniocentesis. The fetal survey was positive for bilateral hydronephrosis, club foot, and VSD with concerns for other cardiac anomalies. A fetal echocardiogram confirmed a complete AV canal defect with a right-sided aortic arch and vascular ring. The patient was also confirmed to have a partial right ureteropelvic junction (UPJ) obstruction.

**Figure 1:**
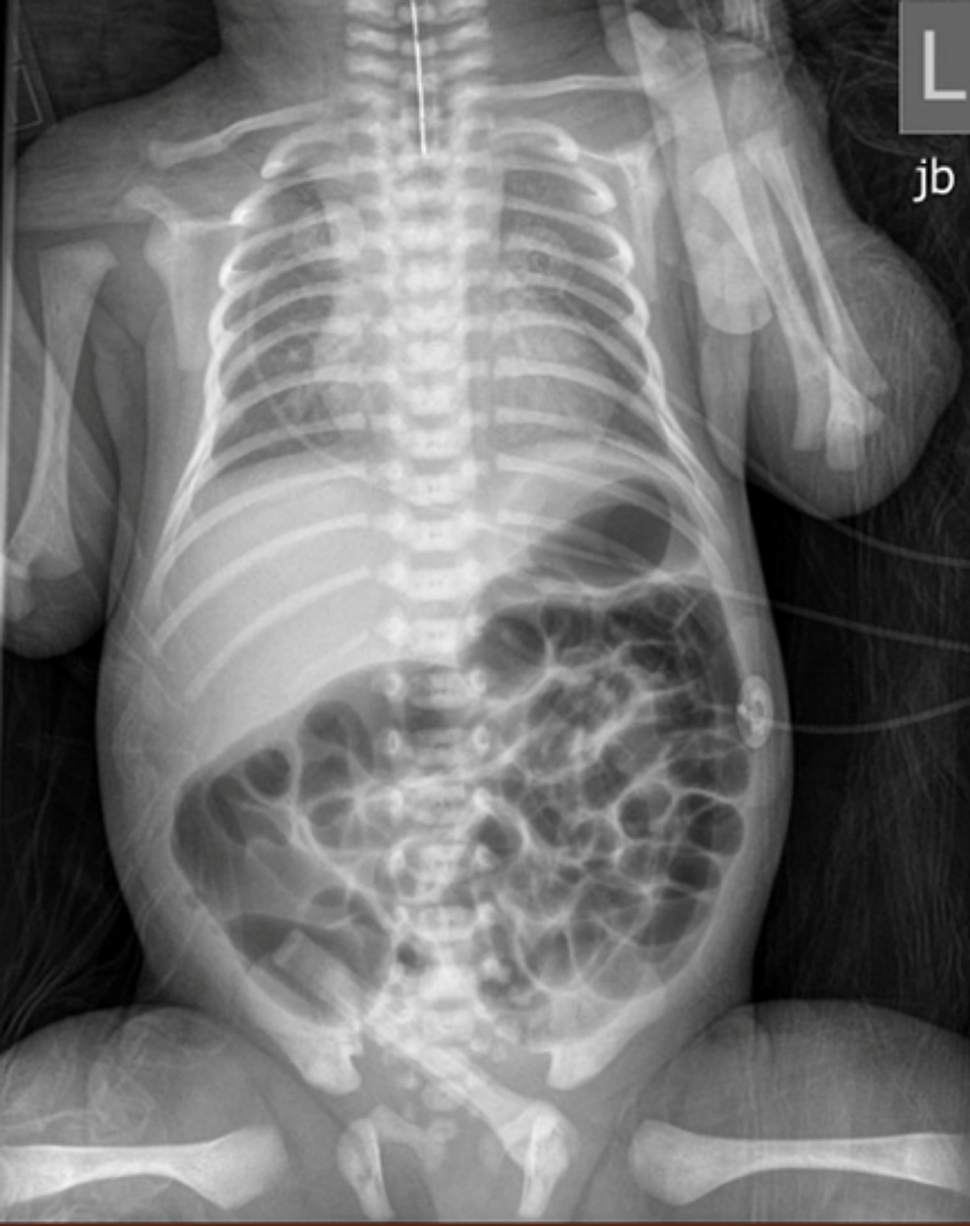
Esophageal atresia with tracheoesophageal fistula. The feeding tube terminates in the proximal esophagus, with bowel gas noted throughout the abdomen. The appearance was suggestive of esophageal atresia with associated tracheoesophageal fistula.

Following delivery at H one on day of life (DOL) one, the patient was admitted to the neonatal intensive care unit (NICU) with concern for EA with TEF after inability to advance an orogastric (OG) tube with curling in the upper chest in the setting of an X-ray with significant air in the stomach and small bowel (Figure 1). The patient was subsequently transferred to a tertiary center on the day of birth for further evaluation and management of his EA/TEF. In addition to the treatment of his additional congenital anomalies, the patient received further VACTERL workup at the tertiary center.

**Figure 2:**
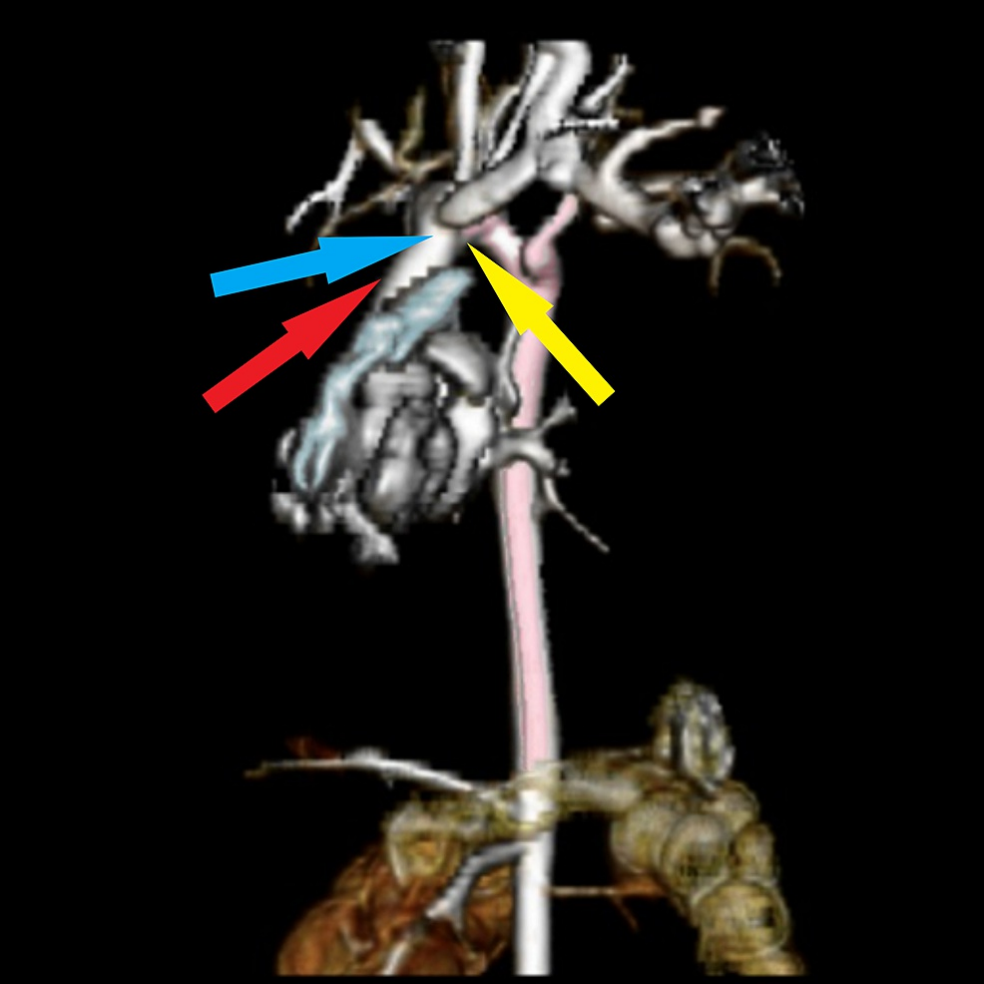
CT chest angiogram. Right-sided aortic arch with left aberrant subclavian artery with a Kommerell diverticulum. Red arrow: right-sided aortic arch; blue arrow: left aberrant subclavian artery; yellow arrow: Kommerell diverticulum.

The patient’s VACTERL workup included renal, spinal canal, head, and cardiac ultrasounds (US). The renal US demonstrated bilateral extrarenal pelvis dilatation with bilateral central calyceal dilation, as well as left proximal ureter dilatation. The spinal canal and head US were normal. The cardiac US revealed a balanced AV canal defect, small atrial septal defect (ASD), moderate VSD, right-sided aortic arch with an aberrant left subclavian artery, and associated vascular ring. A computed tomography (CT) chest angiogram was ordered to better define his vascular anatomy (Figure 2). A timeline of significant interventions to identify and treat the patient’s conditions is described below.

**Figure 3:**
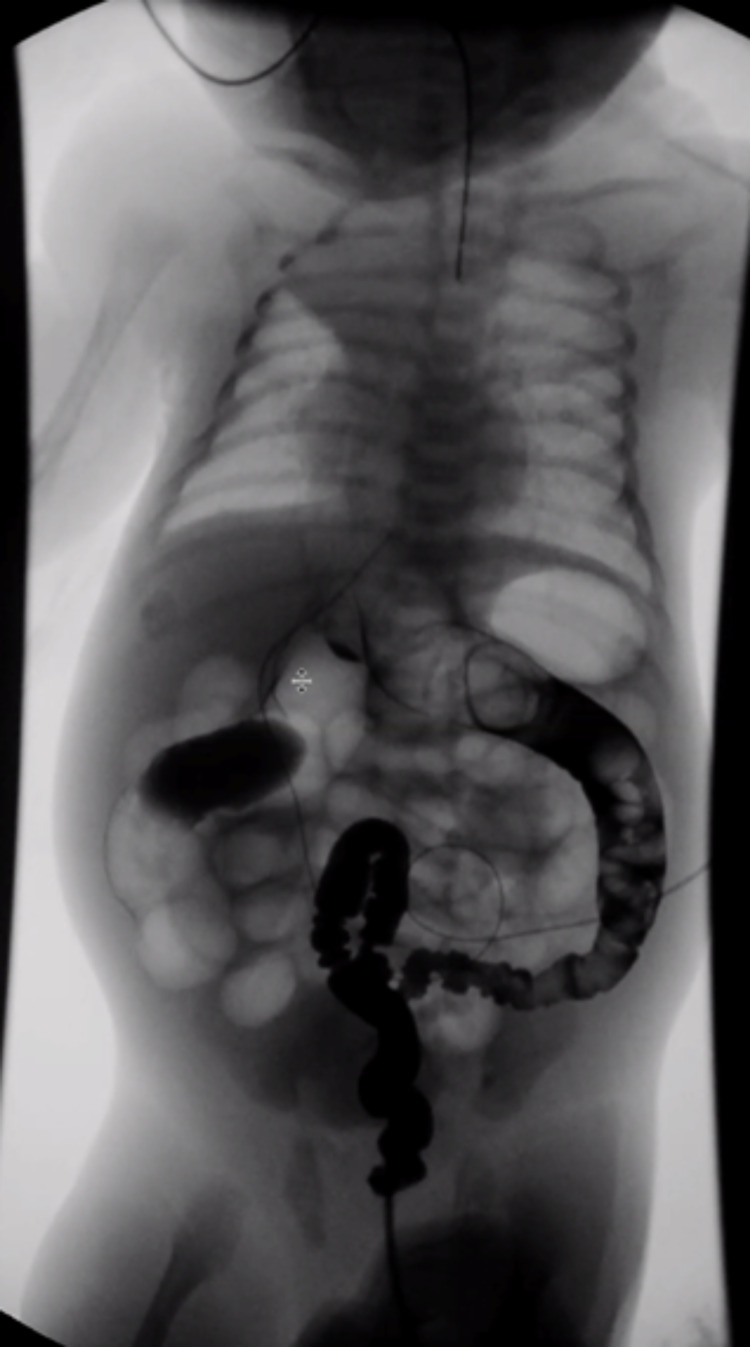
Barium enema suggestive of Hirschsprung's disease. Diffuse small-caliber left colon to the level of the mid transverse colon with saw-tooth irregularity likely representing a long segment of Hirschsprung's disease.

Given the potential for aspiration or pneumonia from saliva spillage in the trachea because of EA/TEF, a Replogle tube was placed in the proximal pouch. In the first 48 hours of life, the patient failed to pass meconium and his abdomen became increasingly distended. Rectal dilation did not produce any stool, and a contrast enema was obtained, which showed a constricted left descending colon and sigmoid colon; both findings were concerning for HD (Figure 3).

On DOL three, he underwent his first operation to confirm the diagnosis with colonic biopsies and diversion. Intraoperative biopsies taken from the sigmoid and transverse colon did not show any ganglion cells but were positive in the terminal ileum; therefore, an end ileostomy with mucous fistula and gastrostomy tube (G-tube) were placed.

On DOL five, rigid bronchoscopy was attempted to repair the TEF, but with complications related to anesthesia, the procedure was postponed. Before the patient was stabilized, he experienced hemodynamic instability and desaturation likely secondary to the massive shunt from the complete AV canal.

On DOL 14, the patient underwent a left posterolateral thoracotomy to repair the congenital EA, tracheoesophageal atresia, and division of his vascular ring. Intraoperative findings indicated a congenital EA with TEF, a right-sided aortic arch with aberrant left subclavian, and a vascular ring. The procedure was completed successfully, and the patient was returned to the NICU in stable condition.

A rigid bronchoscopy on DOL 48 was done to evaluate the status post-EA with TEF repair, as well as to check for any other findings of concern. At this time, a type one laryngeal cleft was identified. Deflux was instilled into the interarytenoid space to build up the mucosa and assist with raising the laryngeal cleft. The procedure was completed successfully, and the patient was returned to the NICU in stable condition.

After experiencing clinical symptoms associated with possible TEF recurrence of unknown etiology, a subsequent bronchoscopy was performed on DOL 97. A large TEF was identified via rigid bronchoscopy, but esophageal dilation was chosen not to be performed. The procedure was concluded, and the endotracheal tube was replaced and successfully positioned beyond the fistula via flexible bronchoscopy.

The patient received surgery on DOL 105 to repair the TEF with a pleural flap. As a result of the procedure, the recurrent TEF was ligated and the trachea and esophagus closed primarily, which was verified by a negative Valsalva leak test. The pleural flap was successfully placed between the trachea and esophagus. The surgical procedure was completed without complication and the patient returned to the pediatric intensive care unit (PICU).

The patient then underwent cardiac repair of his AV canal defect and has recovered well. After prolonged ICU admission, he remains in a pediatric inpatient rehabilitation facility in preparation for transition to home.

## Discussion

Down syndrome, also called trisomy 21, is a genetic disorder characterized by an additional full or partial copy of chromosome 21. DS is the most prevalent genetic disease worldwide and constitutes significant medical and social costs [9]. While DS is known to be associated with several phenotypes, the extent to which these phenotypes display is highly variable. Interestingly, in a large study by Stoll et al. looking at over 728 cases of DS, over 60% of cases of DS had at least one major associated congenital anomaly, in which the most common associated anomalies were cardiac anomalies (44%), followed by digestive system anomalies (6%), musculoskeletal system anomalies (5%), urinary system anomalies (4%), respiratory system anomalies (2%), and other systemic anomalies (3.6%) [10].

While it has been reported that EA with TEF incidence can be associated with additional birth defects and genetic syndromes like DS, VACTERL association workup as a result of multiple organ complications is unique [11]. To our knowledge, our patient represents the first case of confirmed DS diagnosis with several of these rare congenital malformations affecting various organs at birth and the slew of interventions warranted. For the duration of the patient’s care, five surgeries were performed within the first year of life. For patients with DS, their surgical risk is independent of their diagnosis but rather due to their co-morbidities [12]. Thus, it is of utmost importance to determine additional anomalies that may be present. The patient’s early VACTERL association workup, prompted by an early diagnosis of EA with TEF, led to concerning cardiac findings. Additionally, subsequent intervention revealed the presence of HD. Next steps in the management of care were clearly prompted shortly after confirming EA with TEF, which prompted a workup for the VACTERL association that led to the early intervention of several alarming findings. Thus, VACTERL workup in a trisomy 21 newborn with a diagnosis of EA with TEF should be highly considered.

## Conclusions

In this rare case, we report an infant born prematurely at 34 weeks with DS and several anomalies including EA with TEF, laryngeal cleft, vascular ring, HD, atrioventricular canal defect, VSD, right-sided aortic arch with an aberrant left subclavian artery, in addition to bilateral extrarenal pelvis dilatation with bilateral central calyceal dilation, as well as left proximal ureter dilatation. VACTERL workup was prompted because of early diagnosis of EA/TEF and should be considered following confirmed DS diagnosis. To address the constellation of anomalies uncovered, the patient underwent five surgeries before the age of one with additional surgeries necessary. We wanted to share this exceedingly rare case, encouraging a heightened awareness for VACTERL workup in patients with DS.
